# Exploring the cross talk between ER stress and inflammation in age-related macular degeneration

**DOI:** 10.1371/journal.pone.0181667

**Published:** 2017-07-24

**Authors:** Samira Kheitan, Zarrin Minuchehr, Zahra-Soheila Soheili

**Affiliations:** 1 Systems Biotechnology Department, National Institute of Genetic Engineering and Biotechnology, Tehran, Iran; 2 Molecular Medicine Department, National Institute of Genetic Engineering and Biotechnology, Tehran, Iran; University of Pittsburgh, UNITED STATES

## Abstract

Increasing evidence demonstrates that inflammation and endoplasmic reticulum (ER) stress is implicated in the development and progression of age-related macular degeneration (AMD), a multifactorial neurodegenerative disease. However the cross talk between these cellular mechanisms has not been clearly and fully understood. The present study investigates a possible intersection between ER stress and inflammation in AMD. In this study, we recruited two collections of involved protein markers to retrieve their interaction information from IMEx-curated databases, which are the most well- known protein-protein interaction collections, allowing us to design an intersection network for AMD that is unprecedented. In order to find expression activated subnetworks, we utilized AMD expression profiles in our network. In addition, we studied topological characteristics of the most expressed active subnetworks to identify the hubs. With regard to topological quantifications and expressional activity, we reported a list of the most pivotal hubs which are potentially applicable as probable therapeutic targets. Furthermore, we introduced MAPK signaling pathway as a significantly involved pathway in the association between ER stress and inflammation, leading to promising new directions in discovering AMD formation mechanisms and possible treatments.

## Introduction

Age-related macular degeneration (AMD), a multifactorial neurodegenerative retinal disease, impairs the central vision in a significant fraction of over 55 years old population in the world. It has been shown that approximately 8% of the world’s elder population is affected by AMD. The number of people with this disease is anticipated to increase to 196 million by 2020 and to 288 million by 2040 [1].

Numerous studies have focused on pathways and molecular mechanisms involved in the pathogenesis of this ocular disease. The involvement of inflammatory molecules in development and progression of AMD has been investigated in several studies. A possible association between inflammation and AMD was proposed at first by Hageman et al. in terms of the presence of immune response proteins in drusen, which is considered as the most common hallmark in the early stages of AMD [2]. In addition to presence in drusen, multiple genetic polymorphisms in complement elements have been detected in patients with AMD [3–5]. Furthermore, elevated expression in a number of chemokines in different phenotypes of this disease can be considered as a potential link between pro-inflammatory molecules and AMD development [6].

Another biological phenomenon which has been proposed as a key pathogenic mechanism in AMD development is endoplasmic reticulum (ER) stress. ER stress has been proposed as a key pathogenic mechanism in AMD development because of its association with oxidative stress, angiogenesis and apoptosis [7, 8]. Oxidative stress, in which excessive reactive oxygen species (ROS) lead to cellular and molecular impairment, is believed to be a primary cause of damage to the RPE cells. Because of high oxygen consumption and exposure to light in retina, RPE cells are susceptible to the oxidative damage [9]. Inadequately neutralized oxidative stress can lead to oxidation-specific epitopes (OSEs) generation, which can induce immune reaction [10]. In the RPE with AMD, different OSEs, including malondialdehyde (MDA), 4-hydroxynonenal (4-HNE), advanced glycation endproducts (AGE) have been identified [11,12]. Moreover, accumulation of oxidized low density lipoproteins (oxLDL) in Bruch’s membrane can induce a pro-inflammatory response by the RPE [13].

Protein folding is a redox dependent process that leads to ROS generation during disulphide bond formation by protein disulfide isomerase (PDI). Protein folding mediated by PDI in the oxidative environment of the ER become up-regulated under conditions of ER stress. During ER stress, glutathione (GSH) which is the main redox buffer is consumed and redox potential within ER environment becomes increasingly reduced [14]. PDI in its reduced state may act as a chaperone rather than a disulfide isomerase [15, 16]. In response to ER stress in neurodegenerative diseases with protein aggregation, up-regulating of chaperones including PDI protect against misfolded protein accumulation. It has been suggested that PDI participation in initial responses to ER stress is protective, but it may have pro-apoptotic role when proteins are damaged beyond repair [17].

ER stress and inflammation have been linked to a variety of diseases including autoimmune diseases, metabolic disorders and neurodegenerative diseases. Anti ER chaperones antibodies have been recognized in a number of autoimmune diseases such as autoimmune hepatitis [18], rheumatoid arthritis and systemic lupus [19] and inflammatory bowel disease [20]. Genetic inactivation of PERK signaling in multiple sclerosis experimental models exhibit exacerbated experimental autoimmune encephalopathy [21]. In the pathogenesis of metabolic disorders such as type 2 diabetes, it has been shown that ER stress and inflammation are critical contributors to pancreatic β cell dysfunction. ER stress leads to inflammatory cytokine secretion, and these inflammatory cytokines, including IL23, IL24, and IL33, amplify ER stress in pancreatic β cells [22]. It has also been suggested that ER stress and inflammation may contribute to neuronal death in Parkinson’s disease (PD) which is a neurodegenerative disease. Accumulation of α-synuclein, which forms aggregates called Lewy bodies that are characteristic of PD, causes cell death and ER stress [23]. It has been reported that α-synuclein released from Lewy bodies may activate microglial cells and lead to neuroinflammatory responses [24]. In fact, the interplay between ER stress and inflammation in many of these diseases is in the ambiguity. It seems that this interaction is highly dependent on the context of specific diseases and their signaling pathways.

However, although the contribution of inflammation and ER stress in AMD development have been addressed by prior studies, little attention has been paid to clarify the interplay between them. Moreover, as AMD proves to be a complex disease in which several proteins and molecular pathways are involved, traditional “one-gene” approaches would not give us a comprehensive understanding of the disease mechanism. A new conceptual framework has been developed by systems biology which enable us to characterize complex intracellular networks that contribute to cellular functions in normal and pathological conditions [25]. As a major part of interactomes, protein-protein interaction (PPI) networks are powerful tools for decoding the biological process complexity. The biological importance of highly-connected proteins (hubs) in PPI networks occurs because of their involvement in essential complex biological modules [26]. Network analysis using topological properties including degree and in betweenness has provided a powerful tool that can help us identify biomarkers and probable therapeutic targets in neurodegenerative diseases [27].

In this study, we aim to investigate the association between ER stress and inflammation using a systems biology approach in multilateral perspectives. By recruiting the markers of ER stress and inflammation in AMD, we design an intersection network containing the information on how these two biological phenomena are linked. Our ontology analysis indicates that the most enriched pathway associated with this intersection network is MAPK (Mitogen-Activated Protein Kinases) signaling pathway. Furthermore, regarding topological quantifications and expressional activity, we report a list of the most pivotal hubs that the majority of them are MAPK signaling pathway components.

## Materials and methods

### ER stress and inflammation related markers (data set collection)

A literature search was performed to retrieve two lists of ER stress and inflammation markers in AMD. These markers which exhibit differential expression (either at the RNA or protein levels), have genetic variants or existing in drusen, were defined as our seed proteins, Table 1.

**Table 1:** Seed proteins.

Gene Names	Description	References
ER Stress		
HSPA5	Heat shock protein family A (Hsp70) member 5	[28]
ERN1	Endoplasmic reticulum to nucleus signaling 1	[29]
EIF2AK3	Eukaryotic translation initiation factor 2 alpha kinase 3	[29]
ATF6	Activating transcription factor 6	[29, 30]
XBP1	X-box binding protein 1	[31]
ATF4	Activating transcription factor 4	[30]
DDIT3	DNA damage inducible transcript 3	[28, 30]
EIF2A	Eukaryotic translation initiation factor 2A	[30]
Inflammation		
C2	Complement component 2	[4]
C3	Complement component 3	[32]
C5	Complement component 5	[3]
C9	Complement component 9	[3]
VTN	Vitronectin	[33]
CLU	Clusterin	[34]
APP	amyloid beta precursor protein	[35]
CRP	C-reactive protein	[36]
CFH	Complement factor H	[5]
CFD	Complement factor D	[37]
CFB	Complement factor B	[4]
CD46	CD46 molecule, complement regulatory protein	[32]
CR1	Complement component (3b/4b) receptor 1	[32]
CXCL1	C-X-C motif chemokine ligand 1	[6]
CXCL2	C-X-C motif chemokine ligand 2	[6]
CXCL9	C-X-C motif chemokine ligand 9	[6]
CXCL10	C-X-C motif chemokine ligand 10	[6]
CXCL11	C-X-C motif chemokine ligand 11	[6]
CCL2	C-C motif chemokine ligand 2	[6]
CCL8	C-C motif chemokine ligand 8	[6]
HLA-C	Major histocompatibility complex, class I, C	[38]
IL8	Interleukin 8	[39, 40]
IL6	Interleukin 6	[41]
CASP4	Caspase 4	[42]
CASP12	Caspase 12	[43]
TLR4	Toll like receptor 4	[44]
CX3CR1	C-X3-C motif chemokine receptor 1	[45]

### Network construction

PPI information for each protein set were retrieved in International Molecular Exchange (IMEx) consortium members comprising I2D, InnateDB, IntAct, MBInfo, MINT, HPIDB, UCL-BHF, UniProt and MolCon through the PSI Common Query Interface (PSICQUIC)[46]. IMEx-curated databases commit to apply common curation strategies to provide a nonredundant protein-interaction framework (http://www.imexconsortium.org/)[47]. Different types of interaction data which are experimentally determined such as physical association, direct interaction and colocalization have been represented by these databases. Two PPI networks for ER stress and inflammation were visualized using the Cytoscape software (version 3.3.0) [48] and then the intersection network between them was extracted. In a given intersection network, we detected highly interconnected regions (clusters) using Molecular Complex Detection (MCODE) (http://baderlab.org/Software/MCODE). This clustering method finds clusters based on vertex weighting by local neighborhood density and outward traversal from a locally dense seed protein to isolate the dense proteins [49].

### Enrichment analysis

To further understand the biological meaning behind the intersection network, we performed an enrichment analysis using DAVID (Database for Annotation, Visualization and Integrated Discovery), the functional annotation tool, and retrieved Gene Ontology (GO) terms (for more details, see S1 Table). DAVID provides a comprehensive set of functional annotation tools to identify the most pertinent biological processes to a gene/protein set [50]. Using charts of molecular function (GOTERM_MF_FAT), biological process (GOTERM_BP_FAT) and cellular component (GOTERM_CC_FAT), three lists of GO terms and their *p* values were generated independently (for more details, see S2 and S3 Tables). For summarizing and visualizing GO categories, REVIGO (Reduce + Visualizes Gene Ontology) http://revigo.irb.hr/ was applied, with the following parameters: “Small (0.5)” for the allowed similarity and “SimRel” for semantic similarity measure [51]. To gain insight into the most enriched biological pathways of the intersection network and its clusters, the Kyoto Encyclopedia of Genes and Genomes (KEGG) enrichment analysis were also performed.

### Expression data integration

After merging networks of ER stress and inflammation as a united one, expression values were integrated into the network to obtain a more informative network. A comparative transcriptome analysis by Newman et al. is one of the most comprehensive studies on human AMD [6]. GSE29801 with 293 samples from the macular or extra-macular region of normal and AMD human donor eyes was analyzed using the GEO2R (http://www.ncbi.nlm.nih.gov/geo/geo2r/) [52]. GEO2R is an R-based web tool which allows the users to identify and visualize differentially expressed genes and sort by significance using GEOquery and limma packages from the Bioconductor project. After assigning samples to (normal or AMD) a group, *p* value adjustment was applied with the Benjamini & Hochberg false discovery rate method (for more details, see S4 Table). After importing expression data into the network, jActiveModules (version 3.1) was applied to find expression activated subnetworks with significant changes between disease and normal conditions [53]. According to the algorithm of this software, subnetworks were scored using an aggregated Z-score derived from each gene *p* value.

#### Topological analysis

Topological characteristics (degree and betweenness) of highest scored module identified by jActiveModules were examined by CentiScaPe (Version 2.1) [54] to screen for hub proteins. The centrality degree index determines the number of directly connected edges to each node. Nodes with high degree are likely to be a hub having interactions with several other nodes. The betweenness centrality index is calculated by the number of shortest paths passing through a node linking a couple of nodes. The high value of betweenness can indicate the central role of a protein holding together communicating proteins. The values of degree and betweenness for all nodes in the module were plotted in a scatter plot to identify nodes with high values in both the centrality parameters using the Minitab® 17.3.1.

## Results

### Networks of ER stress and inflammation

**Fig 1:**
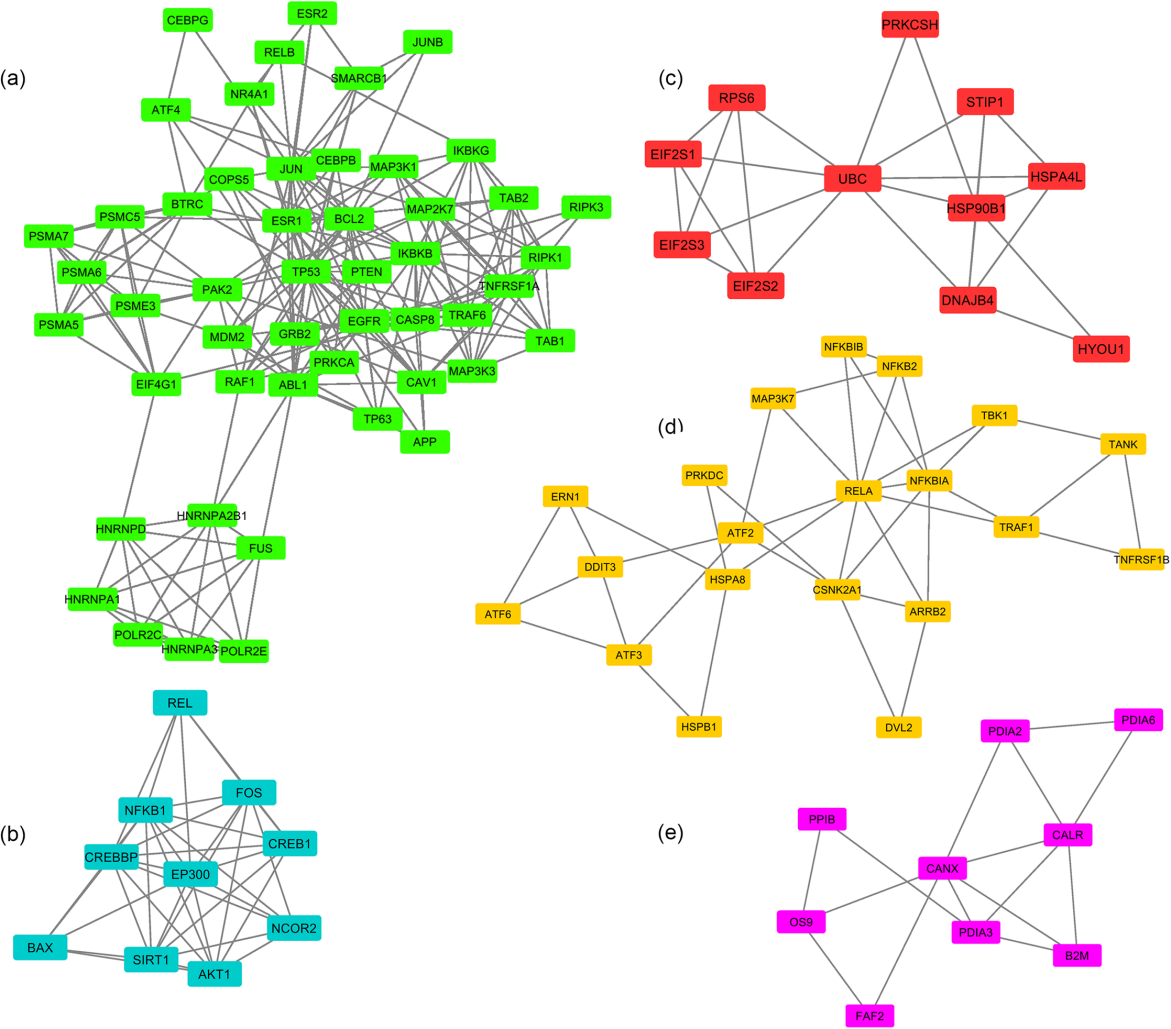
Clusters of intersection network identified by MCODE plugin. a Cluster 1, b Cluster 2, c Cluster 3, d Cluster 4 and e Cluster 5.

PPI data of the known genes of ER stress and inflammation in AMD and their interacting partners were retrieved from IMEx-curated databases and then were applied to construct two separated networks for ER stress and inflammation. The majority of linker proteins in these two networks were obtained from IntAct and MINT databases. Table 2 shows the numbers of nodes and edges derived from IMEx-curated databases in the ER stress and inflammation networks (for more details, see S5 Table). After removing redundant interactions (from organisms other than *Homo sapiens*) with taxid_identifier other than 9606, there were 374 nodes and 666 edges in the ER stress network and 5017 nodes and 16502 edges in the inflammation network. The constructed intersection network between ER stress and inflammation contained 1475 interaction pairs between 269 nodes, (for more details, see S6 Table). Highly connected regions of the intersection network were identified using MCODE plugin implemented in the Cytoscape platform. A total of five clusters as shown in Fig 1 were detected. The number of nodes in clusters 1, 2, 3, 4 and 5 was 50, 10, 11, 20 and 9, respectively (score: cluster 1: 8.98, cluster 2: 8.222, cluster 3: 4.8, cluster 4: 4.105 and cluster 5: 3.75), Table 3. Using DAVID annotation system, we identified the most significant KEGG pathways within each cluster separately, Table 4. The results showed the highest scored cluster (Cluster 1) contains nodes that associate with MAPK signaling pathway (hsa04010).

**Table 2:** PPI data derived from IMEx-curated databases for ER stress and inflammation in AMD.

Databases	Nodes	Edges
ER Stress		
HPIDB	29	41
IntAct	305	510
MINT	66	105
UCL-BHF	4	3
UniProt	7	7
Inflammation		
HPIDB	65	73
I2D-IMEx	62	99
InnateDB-IMEx	69	140
IntAct	3632	10620
MBInfo	8	5
MINT	2418	5038
MolCon	21	33
UCL-BHF	43	62
UniProt	184	432

**Table 3:** MCODE clusters.

MCODE Cluster	Node IDs
1	IKBKB, CASP8, IKBKG, RIPK3, TNFRSF1A, PSMA5, PSMA6, TRAF6, CEBPB, CEBPG, EGFR, PSMA7, RELB, POLR2C, PAK2, POLR2E, MAP3K3, MAP3K1, HNRNPA1, PSMC5, HNRNPA2B1, HNRNPA3, APP, SMARCB1, BTRC, JUNB, TP63, RAF1, PRKCA, PSME3, TP53, ABL1, RIPK1, ATF4, MAP2K7, FUS, CAV1, ESR1, ESR2, NR4A1, JUN, COPS5, MDM2, BCL2, TAB2, TAB1, HNRNPD, EIF4G1, GRB2, PTEN
2	NCOR2, CREB1, AKT1, NFKB1, REL, CREBBP, EP300, BAX, FOS, SIRT1
3	HSPA4L, HSP90B1, EIF2S2, STIP1, EIF2S1, RPS6, UBC, DNAJB4, HYOU1, PRKCSH, EIF2S3
4	TBK1, RELA, ARRB2, NFKB2, ERN1, NFKBIA, CSNK2A1, TNFRSF1B, DDIT3, TRAF1, NFKBIB, DVL2, ATF6, ATF2, ATF3, TANK, HSPA8, PRKDC, HSPB1, MAP3K7
5	PDIA3, PDIA2, OS9, CANX, B2M, PDIA6, CALR, PPIB, FAF2

**Table 4:** KEGG pathways of intersection network and each MCODE cluster by DAVID.

	Number of Nodes	KEGG Pathway	Genes	*p* value
MCODE Cluster 1	50	hsa04010: MAPK signaling pathway	PRKCA, EGFR, GRB2, RELB, TP53, RAF1, NR4A1, TAB1, TAB2, TNFRSF1A, ATF4, PAK2, MAP3K3, MAP3K1, JUN, IKBKG, IKBKB, TRAF6, MAP2K7	6.27E-15
MCODE Cluster 2	10	hsa05161:Hepatitis B	AKT1, FOS, EP300, BAX, CREB1, CREBBP, NFKB1	6.14E-09
MCODE Cluster 3	11	hsa04141:Protein processing in endoplasmic reticulum	HYOU1, HSP90B1, EIF2S1, HSPA4L, PRKCSH	3.95E-05
MCODE Cluster 4	20	hsa05169:Epstein-Barr virus infection	MAP3K7, TRAF1, CSNK2A1, TBK1, RELA, NFKBIB, NFKBIA, HSPB1, NFKB2, HSPA8, ATF2	1.45E-11
MCODE Cluster 5	9	hsa04141:Protein processing in endoplasmic reticulum	PDIA3, PDIA6, CALR, CANX, OS9	1.69E-06

### Enrichment analysis

**Fig 2:**
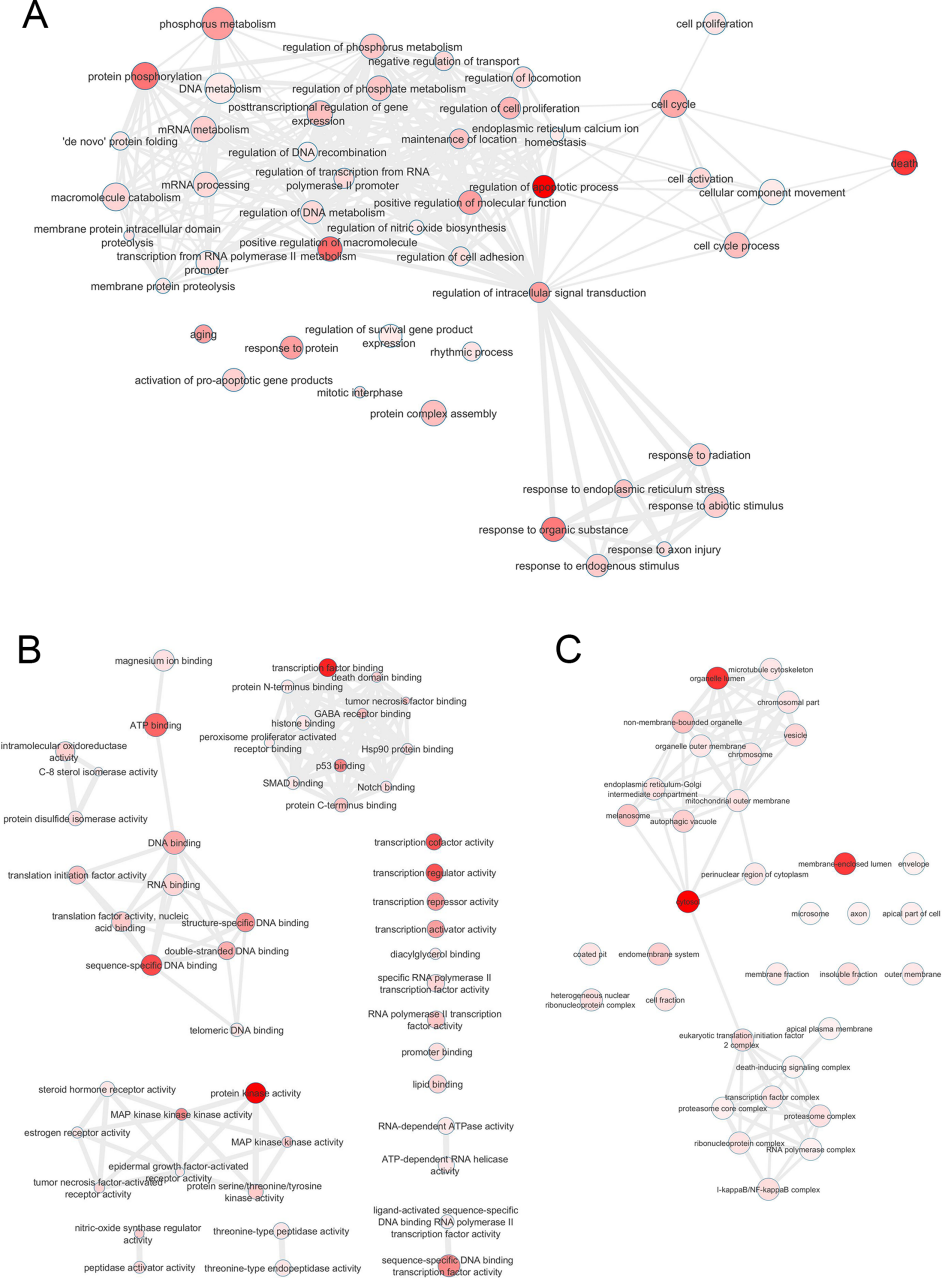
GO terms networks of biological processes (A), molecular functions (B), cellular components (C) and their pvalues associated with shared gene set between ER stress and inflammation. Each node represents a biological process of each gene. Node color indicates the pvalues of each GO term in this intersection (darker = more abundant). Node size indicates the generality of each GO term (smaller = more specific). Edges represent the 3% of the strongest GO term pairwise similarities. The yFiles Organic Layout algorithm was applied to display the topology of the network.

An enrichment analysis was performed on the intersection network in order to identify the most relevant GO terms. GO terms of molecular function, biological process, cellular component and their *p* values were applied to construct three GO networks, Fig 2. GO terms provided by DAVID annotation system with smaller EASE Score (a modified Fisher Exact *p* value) were more enriched and associated with gene list in the intersection. According to the *p* values, the most enriched GO terms of biological process were death (GO:0016265), regulation of apoptotic process (GO:0042981) and protein phosphorylation (GO:0006468), of molecular function they were protein kinase activity (GO:0004672) and transcription factor binding (GO:0008134), of cellular component they were cytosol (GO:0005829), organelle lumen (GO:0043233) and membrane-enclosed lumen (GO:0031974), which are represented in dark red nodes in Fig 2.

### Identification of hubs

**Fig 3:**
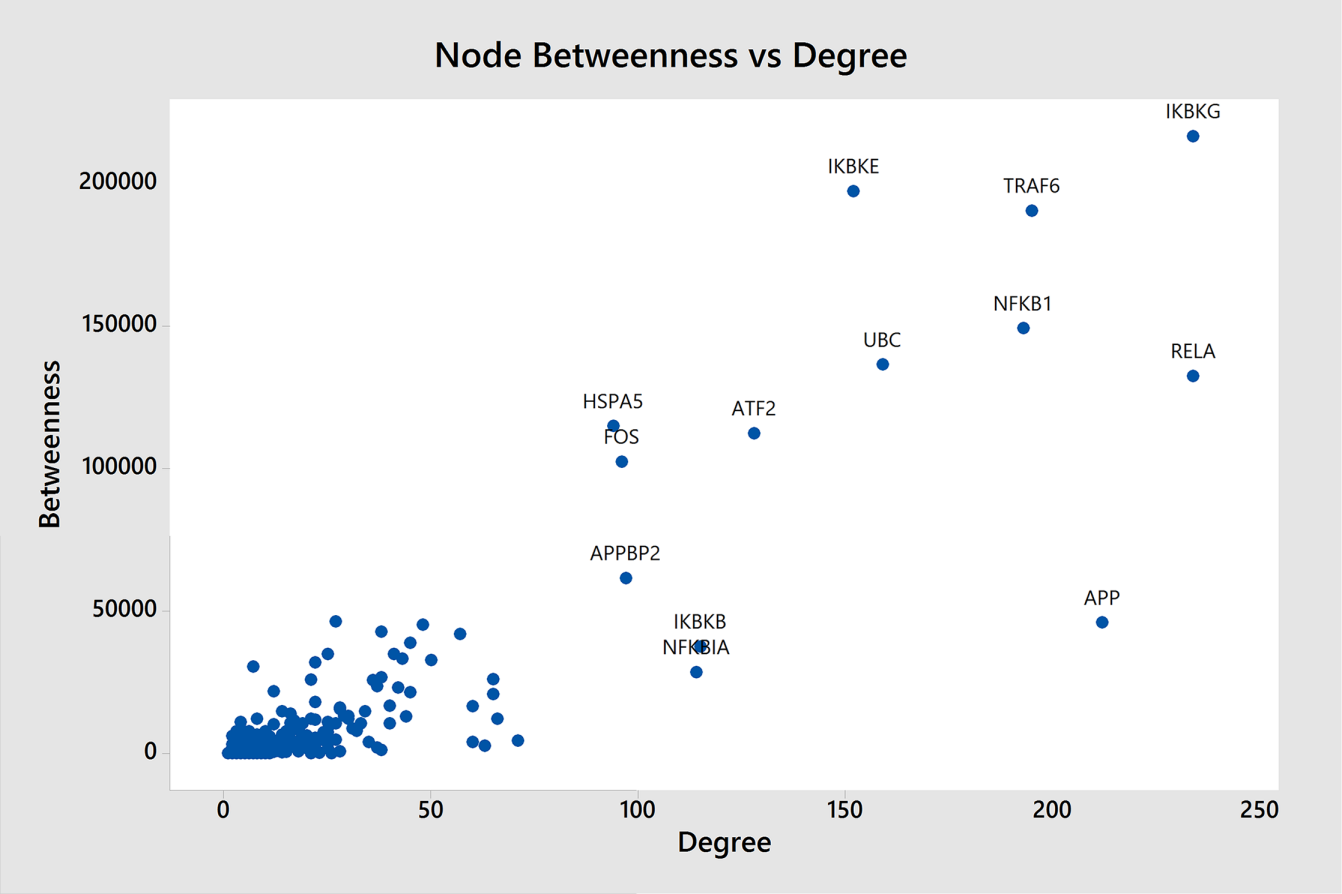
Scatter plot of the centralities parameters.

The Cytoscape plugin, jActiveModules, permits scoring expression activated connected regions of the merged network. All nodes in the most expressed active subnetwork (score: 10.649) consisted of 785 nodes and 4215 edges were examined for centrality parameters (for more detail, see S7 Table). In a scatter plot of two topological parameters, hub nodes with the highest degree and betweenness value were identified, Fig 3 and Table 5 (for more detail, see S8 Table). Inhibitor of kappa light polypeptide gene enhancer in B-cells, kinase gamma (IKBKG), Transcription factor p65 (RELA), Amyloid beta precursor protein (APP), TNF receptor associated factor 6 (TRAF6), Nuclear factor kappa B subunit 1 (NFKB1), Ubiquitin C (UBC), Inhibitor of kappa light polypeptide gene enhancer in B-cells kinase epsilon (IKBKE), Activating transcription factor 2 (ATF2), Inhibitor of kappa light polypeptide gene enhancer in B-cells, kinase beta (IKBKB), NF-kappa-B inhibitor alpha (NFKBIA), Amyloid protein-binding protein 2 (APPBP2), Fos proto-oncogene, AP-1 transcription factor subunit (FOS) and Heat shock protein family A (Hsp70) member 5 (HSPA5) were the hub nodes displaying the highest degree and betweenness within the network. It was noteworthy that seven out of thirteen hubs related to MAPK signaling pathway.

**Table 5:** Hub nodes.

Node Name	Description	KEGG pathway	Betweenness	Degree
IKBKG	Inhibitor of kappa light polypeptide gene enhancer in B-cells, kinase gamma	MAPK signaling pathway	216683.0837	234
RELA	Transcription factor p65	MAPK signaling pathway	132455.8597	234
APP	Amyloid beta precursor protein	Serotonergic synapse	45977.69624	212
TRAF6	TNF receptor associated factor 6	MAPK signaling pathway	190468.8446	195
NFKB1	Nuclear factor kappa B subunit 1	MAPK signaling pathway	149197.6127	193
UBC	Ubiquitin C	PPAR signaling pathway	136502.46	159
IKBKE	Inhibitor of kappa light polypeptide gene enhancer in B-cells, kinase epsilon	Toll-like receptor signaling pathway	197335.8785	152
ATF2	Activating transcription factor 2	MAPK signaling pathway	112390.4689	128
IKBKB	Inhibitor of kappa light polypeptide gene enhancer in B-cells, kinase beta	MAPK signaling pathway	37528.04375	115
NFKBIA	NF-kappa-B inhibitor alpha	cAMP signaling pathway	28564.14077	114
APPBP2	Amyloid protein-binding protein 2	-	61543.76254	97
FOS	Fos proto-oncogene, AP-1 transcription factor subunit	MAPK signaling pathway	102392.7735	96
HSPA5	Heat shock protein family A (Hsp70) member 5	Protein export	114924.3485	94

## Discussion

ER stress and inflammation are common features in AMD pathogenesis and are involved in preserving homeostasis in retina. One of the fundamental questions is that how the two phenomena are linked in this complex ocular disease. In order to explore the cross talk between ER stress and inflammation, this investigation constructed the PPI networks of the two biological phenomena. As far as this team knows, the present study is the first survey to delineate the intersection between ER stress and inflammation using PPI networks, as well as providing clustering and enrichment analysis. Our ontology analysis on intersection MCODE clusters showed that the significantly enriched pathway in the highest scored cluster is the MAPK signaling pathway.

The MAPK signaling pathway regulates the activity of transcription factors using a phosphorylation response to different stimuli [55]. There are three major MAPK pathways, including extracellular regulated kinase (ERK) [56] which respond to growth factors and mitogens, c-Jun NH2-terminal kinase (JNK) [57] and p38 kinase [58] which are activated by a variety of stresses such as UV-exposure, heat shock and ROS. Activation of these pathways is associated with vital diverse downstream processes in cellular fate such as proliferation, differentiation and apoptosis. Imbalance in such processes in RPE cells are the prime early targets for AMD and are involved in the disease development. Induced RPE cell apoptosis by UV exposure [59] and cadmium treatment [60] could be mediated by MAPK signaling pathway so that using specific inhibitors to this pathway may have an effect on reduced RPE cell death. Furthermore, Pons et al. showed the involvement of MAPK signaling pathway in activity of Angiotensin II, the most important associated hormone with hypertension—a potential risk factor for AMD [61].

Moreover, SanGiovanni et al. reported that JNK/MAPK signaling pathway possesses the strongest enrichment signals with identified advanced AMD-associated SNPs [62]. In this study, we have shown for the first time to our knowledge, the most enriched pathway linking ER stress and inflammation in AMD.

Many biological networks such as PPI networks are scale free with the concept that several nodes are linked with a limited number of nodes entitled hubs. Identifying such hubs can lead us to central players in massive and complex networks which are functionally relevant to several proteins. We identified the most significant nodes regarding topological quantifications and expressional activity. Thus, after finding expression activated subnetwork we computed centrality parameters for constituent nodes and eventually a series of hubs were reported. In addition to the two seed proteins, APP and HSPA5, we also found eleven hubs that although the involvement of some of them in AMD pathogenesis had been examined but the participation of others were less well known. In line with our finding which report MAPK signaling pathway as the most enriched pathway associated with ER stress and inflammation intersection, more than half of the detected hubs belong to this pathway.

As parts of IKK (IκB kinase) complex, inhibitor of kappa light polypeptide gene enhancer in B-cells, kinase gamma (IKBKG) and inhibitor of kappa light polypeptide gene enhancer in B-cells, kinase beta (IKBKB) cause nuclear factor kappa B (NF-κB) activation by phosphorylation-induced degradation of inhibitory IκB proteins [63]. Lu H et al. investigated the functional role of IKBKB in the development of laser-induced choroidal neovascularization (CNV), and they have found that IKBKB specific chemical inhibitor significantly reduced the laser-induced CNV formation [64].

Transcription factor p65 (RELA) and nuclear factor kappa B subunit (NFKB1) are Rel-like domain-containing proteins. They are one of the components forming the transcription factor NF-κB. In a study by Diez G et al. [65] on a model for retinal degeneration during iron-induced oxidative stress, an increased level of RELA was observed in nuclear fractions of iron exposed retinas. In the presence of phospholipase A2 inhibitors this overexpression was restored to the control level. Li et al. found that inhibition of apurinic endonuclease 1/redox factor-1 (APE1/Ref-1) redox activity can rescue RPE cells from oxidative stress induced by oxidized low-density lipoprotein (oxLDL) and also lead to reduced level of RELA in oxLDL-challenged RPEs [66].

Fos proto-oncogene, AP-1 transcription factor subunit (FOS) and activating transcription factor 2 (ATF2) are the forming parts of activator protein-1 (AP-1) which is a downstream target of MAPK signaling pathway [67]. Impacted by its components and cellular context, this transcription factor contributes to different biological processes such as light induced apoptotic cell death of photoreceptors [68].

TNF receptor associated factor 6 (TRAF6) is the other hub found, that has been implicated in NF-κB and MAPK pathways activation in response to the signals from receptor families such as TNF receptor superfamily and IL-1R/Toll-like receptor superfamily. As E3 ubiquitin ligase, TRAF6 propagates signals mediating the synthesis of K63-linked-polyubiquitin chains [69]. Ubiquitin C (UBC) is one of the three eukaryotic gene families encoding ubiquitin [70]. Its conjugation into the target proteins can be as a monomer or a polymer linked by different Lys of the ubiquitin. Based on the linking Lys residues, their attachment to a target protein leads to different cellular destinations such as lysosomal degradation, DNA repair, endocytosis and ERAD [71]. Amyloid protein-binding protein 2 (APPBP2) is involved in controlling post-transcriptional mechanisms including mRNA decay pathway [72]. It has reported that translocation of APP along microtubules to the basolateral surface is associated with interacting with APPBP2 [73].

The list of the most pivotal hubs including newly introduced and already investigated ones that we presented in this study can be applied as a complete and practical set of probable therapeutic targets. In contrast to global biomarkers that have relied upon only expression differences, in this study we couple expressional activity with topological characteristics which enable us to introduce key players in AMD more accurately and comprehensively.

A more complete delineation of the underlying cellular and molecular mechanisms involved in a complicated pathophysiologic process of AMD is required for navigating into novel therapeutic targets. In this study, we presented the association between ER stress and inflammation—in terms of biological networks—exclusively in AMD. Our results indicated that MAPK signaling pathway and its components have been the most involved players in this communication. The list of the most pivotal hubs including newly introduced and already investigated ones that we presented in this study can be applied as a complete and practical set of probable therapeutic targets.

MAPK signaling pathway has also recently become a spotlight in clinical studies of cancer toward the discovery of new drug targets. In fact, it is estimated that approximately one third of human cancers are affected by mutations in components of this pathway. Drug targeting members of MAPK signaling pathway including RAF proto-oncogene serine/threonine-protein kinase (RAF1) and mitogen-activated protein kinase kinase 1 (MAP2K1), which were also presented in our intersection network, are ongoing projects leading to substantial progress [74].

Today, intravitreal vascular endothelial growth factor (VEGF) inhibitors are the main drugs for AMD treatment. By binding free circulating VEGF or its receptors, these inhibitors prevent neovascularization. New anti-VEGF agents including brolucizumab [75], conbercept [76], designed ankyrin repeat protein (DARPin) [77] and sFLT01 [78] are currently under investigation. Despite significant visual improvements in patients with AMD since the availability of new drugs, challenges in AMD treatment are still present. It has been reported that patients with refractory or recurrent AMD may develop mechanisms of resistance, especially to anti-VEGF therapy [79]. In fact, the multifactorial pathogenic mechanism of AMD might explain the insufficient response to the current drugs. Therefore, targeting alternative contributions from different pathways is needed.

Computational exploration of major players in AMD pathogenicity is still an open challenge in etiological studies of the disease. The results of the present study shows the major role of MAPK signaling pathway as an intersectional role in both ER stress and inflammation of AMD underlying promising new directions to mechanism discovery and the treatment of AMD.

## Supporting information

S1 Table Enrichment analysis input. (XLSX)

S2 Table GO terms. (XLSX)

S3 Table REVIGO input. (XLSX)

S4 Table Expression dataset. (XLSX)

S5 Table Loci derived from IMEx databases. (XLSX)

S6 Table Intersection network. (XLSX)

S7 Table The most expressed active subnetwork. (XLSX)

S8 Table Topological parameters. (XLSX)
